# Wildlife road traffic accidents: a standardized protocol for counting flattened fauna

**DOI:** 10.1002/ece3.1097

**Published:** 2014-07-10

**Authors:** Wendy J Collinson, Daniel M Parker, Ric T F Bernard, Brian K Reilly, Harriet T Davies-Mostert

**Affiliations:** 1 The Endangered Wildlife TrustJohannesburg, South Africa; 2 Wildlife and Reserve Management Research Group, Department of Zoology and Entomology, Rhodes UniversityGrahamstown, South Africa; 3 Department of Nature Conservation, Tshwane University of TechnologyPretoria, South Africa

**Keywords:** Detection, experimental trials, modeling, protocol, road transects, roadkill, species richness, wildlife traffic mortality

## Abstract

Previous assessments of wildlife road mortality have not used directly comparable methods and, at present, there is no standardized protocol for the collection of such data. Consequently, there are no internationally comparative statistics documenting roadkill rates. In this study, we used a combination of experimental trials and road transects to design a standardized protocol to assess roadkill rates on both paved and unpaved roads. Simulated roadkill were positioned over a 1 km distance, and trials were conducted at eight different speeds (20–100 km·h^−1^). The recommended protocol was then tested on a 100-km transect, driven daily over a 40-day period. This recorded 413 vertebrate roadkill, comprising 106 species. We recommend the protocol be adopted for future road ecology studies to enable robust statistical comparisons between studies.

## Introduction

South Africa is the third most biologically diverse country (Bartels and Kotze 2006; IUCN Red List 2012), the 25th largest country in the world and ranks 18th in terms of total road length (65,600 km paved, 689,000 km unpaved) and 74th for the number of cars per 1000 people (123/1000; CIA 2012). With vertebrates coming under increasing pressure from human development (Dodd and Smith 2003), the demand for a quick, reliable, and statistically robust method of recognizing the latent threat of roads is increasingly urgent (Erritzøe et al. 2003).

Globally, there is increased scientific interest in roadkill and road ecology (e.g., Seiler 2004; Sutherland et al. 2010), but very few studies have been conducted in South Africa, even though roadkill has the potential to significantly affect biodiversity (Bartels and Kotze 2006). Previous assessments of wildlife road mortality detection methods have differed (Santos et al. 2011) and are thus not directly comparable (Evink 2002; Erritzøe et al. 2003). There is, therefore, a need to investigate the factors influencing the detection of roadkill for a wide variety of species (Erritzøe et al. 2003; Ford and Fahrig 2007; Kolowski and Nielsen 2008) and to develop a standardized protocol to estimate roadkill rates. This will enable longitudinal study in same-site areas allowing trends to be monitored. Furthermore, international comparative statistics of roadkill can be documented (Shyama Prasad Rao and Saptha Girish 2007).

Here, we assess the impact of speed driven, size of roadkill, the position of the sun, and whether the observer is the driver or passenger on the detection of roadkill and present a standardized protocol for the assessment of roadkill. In addition, we provide a model for future roadkill sampling in areas of high and low species richness and propose minimal durations (i.e., number of sampling days required) and distances (i.e., transect length; km).

### Study area

**Figure 1:**
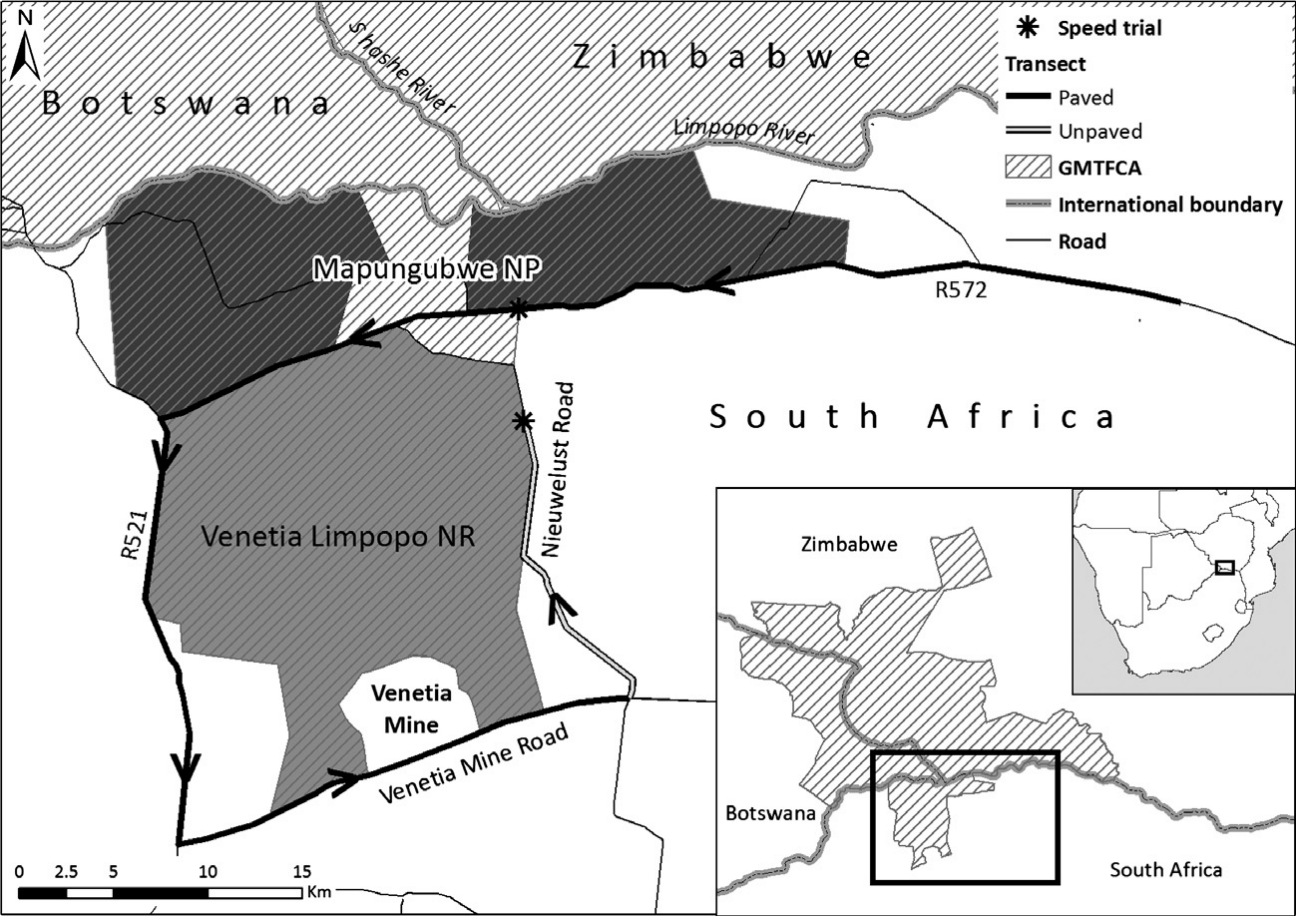
A map of the Greater Mapungubwe Transfrontier Conservation Area (GMTFCA), northern Limpopo (South Africa), showing the roads sampled during the experimental methods. *Indicates the two locations of the 1-km speed trials on the paved and unpaved road. The 100-km transect road is highlighted in bold, showing the R572 national highway (57.1 km), the R521 national highway (23.7 km), the Venetia Mine Road (19.2 km), and the Nieuwelust unpaved road (23.1 km).

The study was carried out in the Greater Mapungubwe Transfrontier Conservation Area (GMTFCA) in the Limpopo Province of South Africa (Fig. 1). The GMFTCA has a high species richness for reptiles (120 species; Branch 1998), birds (at least 429 species; Hockey et al. 2005), and mammals (about 100 species; Skinner and Chimimba 2005) and a lower species richness for the amphibians (about 12 species; Braack 2009).

## Materials and Methods

### Meta-analysis of previous studies and methods

A Google Scholar search using the term “vertebrate roadkill survey” provided 1450 results. The first 10 pages were reviewed to locate peer-reviewed journal articles which assessed roadkill detection. We did not constrain the search by geographic location or to cover a particular period and located 61 peer-reviewed roadkill studies that monitored roadkill from a vehicle. For each of the 61 studies, we extracted the following information: speed driven, when transects were driven, direction travelled, number of observers, sampling distance, frequency, and duration of sampling (Appendix S1) with each of these summarized as a mean and median (Table 1).

**Table 1:** A summary of the 61 peer-reviewed studies showing the mean and median speeds driven, the number of observers used, the sampling frequency, the mean sampling distance, and the number of transects driven (taken from a search on Google Scholar using the words “vertebrate roadkill survey”).

Technique	*n* of 61 studies	Mean/median	Range
Mean speed (km·h^−1^)	28	53	15–100
Median speed (km·h^−1^)	28	51	–
Mean/median observers	21	2	1–2
Mean sampling frequency (months)	47	29	4 days–168 months
Median sampling frequency (months)	47	24	–
Mean sampling distance (km)	40	185.2	1.6–732
Median sampling distance (km)	40	41.8	–
Mean number of transects	42	4	1–15
Median number of transects	42	2	–

### Experimental methods

#### Speed trials

We used speed trials to determine the maximum speed at which to detect roadkill. Twenty simulated roadkill were fabricated from squares of painted rubber sprinkled with sand and gravel to resemble flattened carcasses. Two sizes, large roadkill (*n* = 10), simulating a large bird roadkill (e.g., Swainson's Spurfowl *Pternistes swainsonii*), and small roadkill (*n* = 10), simulating a small rodent roadkill (e.g., Bushveld gerbil *Tatera leucogaster*), were used. The simulated roadkill were placed at randomized points (generated using RNG, Microsoft Office Excel 2010, Microsoft Corporation, Redmond, WA, USA) on a 1–km-long straight, single-carriageway road (Fig. 1). A 1-km transect was selected to minimize observer fatigue and driver safety. The road (mean width = 6 m) was separated into seven zones, each one meter apart. Zone 0 was located at the left-hand verge, with Zone 3 being the center of the road, and Zone 6 on the right-hand verge. The stretch of road was selected as visibility of the road and simulated roadkill were not impeded by shadows cast by trees on the roadside or raised verge sides. No rain was recorded during the speed trials therefore eliminating possible water reflection on the road surface (Konstantopoulos et al. 2010).

The 1-km transect was driven 15 times at each of the following speeds: 20, 30, 40, 50, 60, 70, 80, and 100 km·h^−1^. Simulated roadkill (*n* = 20) were re-positioned after each 1 km traverse, and the position along the transect and the zone were randomized. Two methods of counting traffic were employed (observational and sensor techniques) to assess potential disruption to other drivers and ensure the safety of the researchers during the trials (Collinson 2013). Traffic intensity was low (Seiler 2005) with an average-daily-vehicle usage of 200 vehicles per 24 h on the paved road and 15 vehicles per 24 h on the unpaved road (Collinson 2013).

The same three researchers conducted all of the trials, two laid out the course and collected the simulated roadkill and the third (WC) observed the roadkill. In order to establish whether there was a difference in detection rate with the same observer as the driver or as the passenger (Clevenger et al. 2003; Barrientos and Bolonio 2009), the full set of transects was repeated with “driver-as-the-observer” and “passenger-as-the-observer.” In these cases, the observer was the principal researcher and was considered an experienced observer. As it is unlikely that an untrained observer would be used, we compared roadkill detection at a wider range of vehicle speeds (20, 30, 40, 50, 60, 70 80, and 100 km·h^−1^) using the driver alone as the experienced observer and the passenger as the experienced observer.

To examine the need for the observer to be experienced, an inexperienced observer as the passenger completed one trial at three different speeds (20, 60, and 100 km·h^−1^). A scribe recorded either “large” or “small” as roadkills was observed during each replicate for each observer type (*n* = 3). This procedure was repeated on a 1-km stretch of unpaved road at speeds of 20, 40, and 60 km·h^−1^ because 60 km·h^−1^ is the maximum speed limit on unpaved roads in South Africa (The Road Traffic Act 1989).

Speed trials were conducted at different times of the day (from dawn to dusk) and driven from east-to-west and west-to-east to assess whether light conditions and the angle of the sun affected detection. Three categories of light condition were identified as “sun in eyes” (driving east up to three hours after dawn/driving west up to three hours before sunset), “sun behind” (driving east up to three hours before dusk/driving west up to three hours after dawn), and “sun above” (driving east or west more than three hours after dawn and more than three hours before dusk). A right-hand-drive vehicle was used with the road driven on the left-hand side of the roadway, according to South African driving regulations.

#### Field transects

Field data were collected (using the protocol emerging from the speed trials described above) during the hot/wet season (February–May) (Viljoen et al. 2008), which is when vertebrate species are most active and when migratory species were most likely to be present (Skinner and Chimimba 2005; Carruthers and Du Preez 2011).

To assess the optimal distance and duration for the sampling required to adequately assess roadkill rates, a 100-km paved road transect (Fig. 1) was driven for 40 consecutive days (in March 2012). One observer (the driver) conducted this survey, and the transect was driven at speeds of between 40–50 km·h^−1^. The same direction was driven each day, traveling anticlockwise (Fig. 1). For each carcass, a photograph was taken, and the position on the road and a GPS position (using a Garmin eTrex 10, Garmin Ltd., Olathe, KS, USA) recorded to avoid recounts on consecutive days.

### Statistical procedures

#### Speed trials (1-km transects)

The influence of speed on the detection of simulated roadkill was tested using a two-way ANOVA (STATISTICA, version 10, StatSoft, Inc. Tulsa, OK 2011), and the results were considered significant at *P* < 0.05 (Fowler et al. 2009). Speed and road surface type (paved/unpaved) were categorical independent variables, and the number of simulated roadkill detected per speed category, the dependent variable. A Scheffé's post hoc range test was used to examine differences among means when tests were significant.

A two-way ANOVA was used to test whether vehicle speed and the three observer types influenced roadkill observation at different speeds (20 and 100 km·h^−1^).

To examine the influence of light condition on detection rate, speeds were pooled into two categories (slow, 20–50 km·h^−1^ and fast, 60–100 km·h^−1^). A mean detection rate for speed was generated for each category and was tested in a two-way ANOVA with vehicle speed and light condition as categorical independent variables.

A two-way ANOVA was used to assess whether the position of the roadkill (zone) on the road and speed influenced roadkill detection, with speed (pooled as above) and zone as categorical independent variables.

#### Field transects (100-km transect)

The optimal distance and duration of sampling were determined using species accumulation curves for each vertebrate group (amphibians, reptiles, birds, and mammals) using EstimateS 9.0 (Colwell 2013). The observed species richness (Mao Tau) (Magurran and Queiroz 2010) from field transects was used to construct species accumulation curves for each taxonomic group (Chazdon et al. 1998; Magurran and Queiroz 2010). Adequate sampling was defined as the point where the estimated richness was equal to or less than the richness observed by daily sampling (Gotelli and Colwell 2001; Magurran and Queiroz 2010).

We modelled the effect of species richness (low, medium, and high) for each vertebrate group and estimated optimal durations and transect lengths. Two species richness estimators, Chao 2 (Chao 1987) and ICE (Incidence-based Coverage Estimator) (Chao et al. 2009), were calculated for each vertebrate group and compared to the observed species richness (Mau Tao). We modelled on the assumption that species richness was high in the GMTCA for reptiles, birds, and mammals, and low for amphibians (Braack 2009).

To estimate the sampling effort required in an area of intermediate diversity (for reptiles, birds, and mammals), the number of species in the EstimateS data matrix was reduced by 50% through removing the lower half of the data matrix. For low species diversity, the number of species in the data matrix was reduced by a further 50% by removing the lower half of the data matrix.

To obtain a measure of adequate sampling effort for Amphibia in an area of intermediate species diversity, the number of species in the EstimateS data matrix was increased and resampled by 50% (i.e., from two to four species) using the resampling function tool in Microsoft Office Excel (2010). Similarly, data were increased and resampled by a further 50% (i.e., from four to eight species) for high amphibian diversity.

## Results

### Meta-analysis of previous studies and methods

The majority of the 61 previous roadkill assessment studies took place in North America (38%) with 33% in Europe, 11% in South America, 7% in Australia/New Zealand, and 7% in southern Africa. Three studies were from other countries. Most studies (83%) were conducted after 2000, with only 10% conducted between 1980 and 1999, with the remaining 7% before 1980. A majority (*n* = 44) provided limited or no information on how the studies were carried out (Appendix S1). Surveys were conducted on a range of road types, from highways to unpaved roads. Different speeds were driven, as well as at different times of the day (Appendix S1). The average speed was 53 km·h^−1^ (*n* = 28, range 15–100 km·h^−1^; Table 1), and the frequency, duration, and transect lengths varied considerably (Table 1).

Santos et al. (2011) suggested that the most accurate method of sampling roadkill was to sample “on foot,” although Guinard et al. (2012) noted that roadkill surveys by vehicle were as efficient as surveys by foot although less efficient for carcasses on verges. Our protocol examines only roadkill surveys conducted from a vehicle because these were the most abundant survey methods noted during the meta-analysis.

### Speed trials

#### Vehicle speed, road surface type, and simulated roadkill size

**Figure 2:**
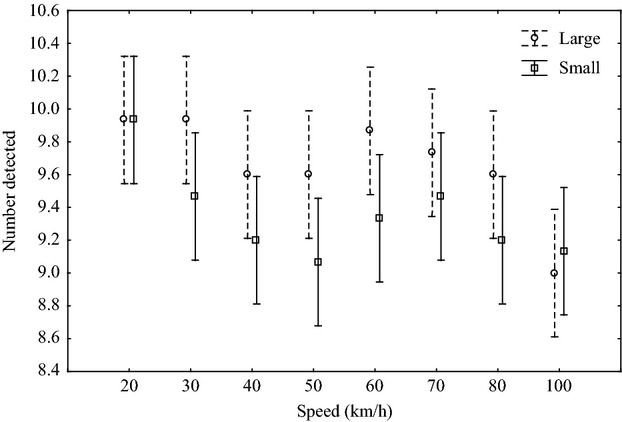
The mean (±0.8; 95% CI) number of large and small simulated roadkill detected at eight speeds during experimental testing along a 1-km section of paved road in the GMTFCA, South Africa.

**Figure 3:**
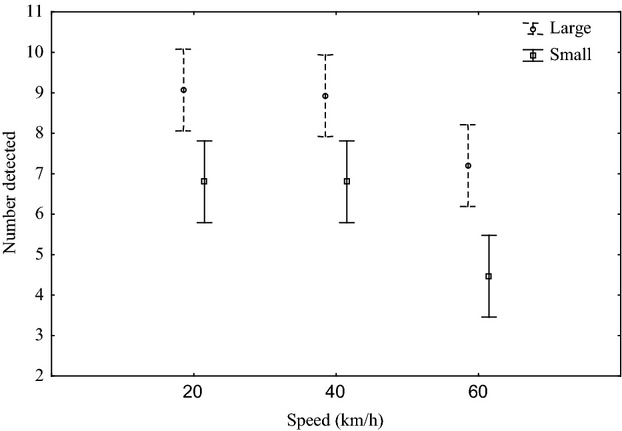
The mean (±2.5; 95% CI) number of large and small simulated roadkill detected at three speeds during experimental testing along a 1-km section of unpaved road in the GMTFCA, South Africa.

Simulated roadkill detection was significantly affected by the vehicle speed (*F*_7,224_ = 03.55, *P* < 0.05; Fig. 2) and by simulated roadkill size on paved roads (*F*_1,224_ = 5.7, *P* < 0.05; Fig. 2). Maximum detection rates were encountered at 20 km·h^−1^ (19.9 ± 0.4), decreasing in accuracy as speed increased to 30 (19.4 ± 1.2), 40 (18.8 ± 0.9), and 50 km·h^−1^ (18.7 ± 0.7), respectively. A slight increase in detection was observed at speeds of 60 (9.2 ± 0.9) and 70 km·h^−1^ (19.2 ± 1.3), but decreased at 80 (18.8 ± 1) and 100 km.h^−1^ (18.1 ± 1.6). Speed and body size did not interact to influence detection (*F*_7,224_ = 0.8, *P* = 0.6; Fig. 2), and detection rates were lower at 100 km·h^−1^ (9.1 ± 1.1) than at 20 km·h^−1^ (9.9 ± 0.3) (*F*_7,232_ = 3.4, *P* < 0.05; Fig. 2). Speed and body size influenced detection on the unpaved road (speed; *F*_2,84_ = 10.6, *P* < 0.05; size; *F*_1,84_ = 32.9, *P* < 0.05; Fig. 3), although there was no interaction between the two variables (*F*_2,84_ = 0.1, *P* = 0.9; Fig. 3). Detection on the unpaved road was lower at 60 km·h^−1^ (5.9 ± 2.8) than at either 20 (7.9 ± 1.7) or 40 km·h^−1^ (7.9 ± 1.9) (*F*_2,87_ = 7.8, *P* < 0.05; Fig. 3).

#### Vehicle speed and observer skill

**Figure 4:**
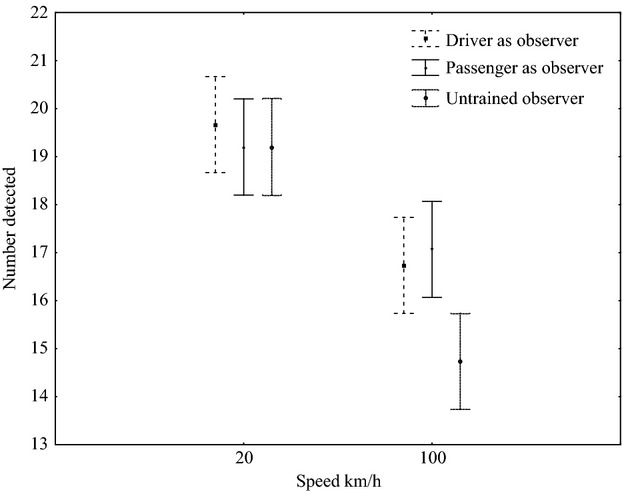
The influence of observer experience and speed on the detection of simulated roadkill at two speeds during experimental testing along a 1-km section of paved road in the GMTFCA, South Africa. Data are means (±2.6; 95% CI) for both large and small roadkill.

**Figure 5:**
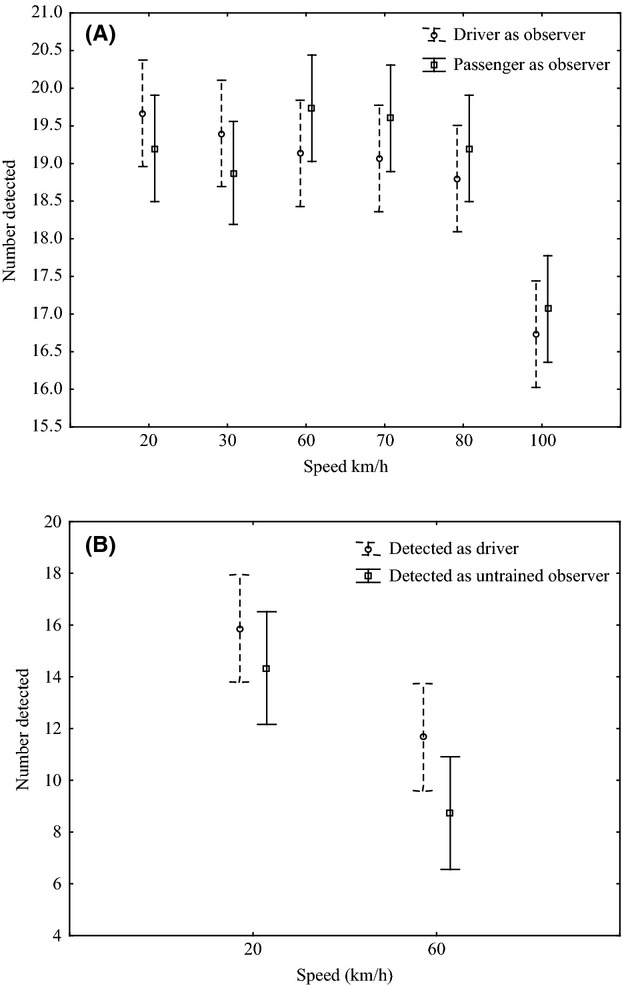
The influence of speed and observer experience on the detection of simulated roadkill along a 1-km section of (A) paved and (B) unpaved roads in the GMTFCA, South Africa. Data are means (±95% CI) for both large and small roadkill (A) the difference between driver and passenger detection rates (at six vehicle speeds) (±1.6) and (B) the difference between driver experience (with two different observers) and detection rates at two speeds (±2.8).

Speed and observer type influenced detection on the paved road (*F*_1,84_ = 59.8, *P* < 0.05 and *F*_2,84_ = 3.8, *P* < 0.05, respectively; Fig. 4). However, there was no interaction between the two variables (*F*_2,84_ = 2.8, *P* = 0.07). At 20 km·h^−1^, there was no difference in the detection among the three observer types (driver as the observer 19.8 ± 0.8; passenger as the observer 19.2 ± 0.9; inexperienced observer 19.2 ± 1.2), (Fig. 4). The untrained observer detected fewer roadkill at 100 km.h^−1^ (14.7 ± 2.4) than either of the trained observers (driver as the observer 16.7 ± 2.9; passenger as the observer 17 ± 2.3) (Fig. 4). All observers detected fewer simulated roadkill at 100 km·h^−1^ than at 20 km·h^−1^ (*F*_1,84_ = 59.8, *P* < 0.05; Fig. 4). Detection was not influenced by whether the observer was the driver or the passenger on paved (*F*_5,169_ = 1, *P* = 0.41; Fig. 4) or unpaved roads (*F*_1,168_ = 0.49, *P* = 0.5); however, all observer types detected fewer simulated roadkill at 100 km·h^−1^ than at slower speeds (*F*_5,168_ = 14.9, *P* < 0.05; Fig. 5A). There was no significant interaction between vehicle speed and observer type on the paved road (*F*_5,168_ = 1, *P* = 0.4; Fig. 5A); however, the inexperienced observer detected significantly fewer simulated roadkill than the experienced observer at both 20 (experienced observer 15.9 ± 4.5; inexperienced observer 14.3 ± 4) and 60 km·h^−1^ on the unpaved road (experienced observer 11.6 ± 5.1; inexperienced observer 8.7 ± 4.2) (*F*_2,27_ = 13, *P* < 0.05; Fig. 5B).

Speed and observer type also affected the accuracy with which simulated roadkill were correctly identified as large or small. At speeds between 70 and 100 km·h^−1^, 0.5% (*n* = 900) of large simulated roadkill were misidentified as small. The inexperienced observer not only misidentified the size but also counted extra “objects” on the road as roadkill. The inexperienced observer detected an extra 0.6% roadkill when driving at 20 km and this increased to 1.3% at 50 km·h^−1^.

#### Vehicle speed, light conditions, and zone

Sun position had no significant effect on detection of simulated roadkill (*F*_2,18_ = 0.7, *P* = 0.5) (number detected: sun above 97%; sun in eyes 95%; sun behind 93%). Similarly, detection of simulated roadkill was not affected by its position on the road (zone), irrespective of size (small = *F*_40,2_ = 6.3, *P* = 0.14; large = *F*_40,2_ = 6.7, *P* = 1.4).

#### Speed trials summary

Based on the speed trials, field transects were driven with a single experienced observer as the driver, at between 40 and 50 km·h^−1^ (because this was the maximum speed that could be driven before detection became less accurate) starting at 1.5 h after dawn and ending no later than 1.5 h before dusk.

#### Field transects

Over 40 days and 122.1 hours (average = 3.1 h per day), 4000 km were driven. A total of 413 individual roadkill were observed on the 100-km transect over the 40-day sampling period, comprising 106 species from all terrestrial vertebrate groups (Appendix S3).

#### Species richness versus duration (number of days) and distance (km)

**Figure 6:**
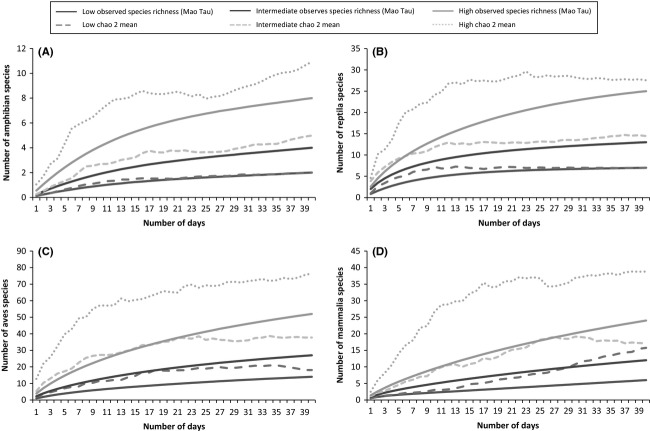
Species accumulation curves showing modelled high, intermediate, and low species richness for the four vertebrate taxonomic groups (A, Amphibia, B, Reptilia, C, Aves, and D, Mammalia) and sampling frequency for days (over a 40-day period per 100-km transect in the GMTFCA. Adequate sampling was defined as the point where the estimated richness was equal to or less than the richness observed (Mao Tau) by daily sampling. Chao2 (as the more conservative estimator) is displayed in all three models.

**Figure 7:**
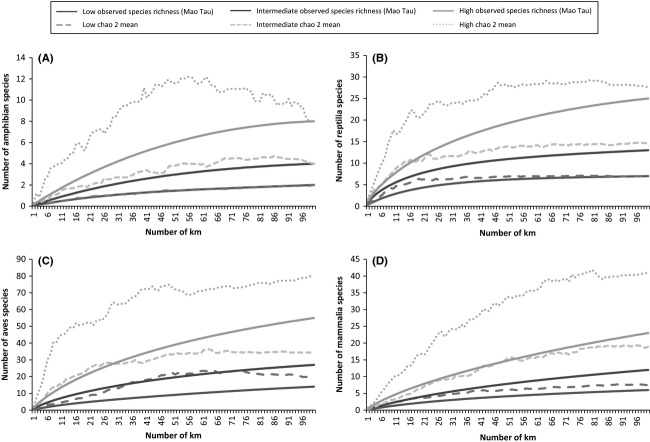
Species accumulation curves showing modelled high, intermediate, and low species richness for the four vertebrate taxonomic groups (A, Amphibia, B, Reptilia, C, Aves, and D, Mammalia) and sampling frequency for km (over a 40-day period per 100-km transect in the GMTFCA. Adequate sampling was defined as the point where the estimated richness was equal to or less than the richness observed (Mao Tau) by daily sampling. Chao2 (as the more conservative estimator) is displayed in all three models.

Based on a transect distance of 100 km over a 40-day period, the mean sampling duration required to achieve adequate sampling for the two estimators (ICE and Chao 2) was calculated for the three modelled levels of species diversity for each taxonomic group (Table 2). Using the most conservative estimator (Chao 2), mammals required the greatest sampling frequency for all three diversity levels (Table 2; Fig. 6D). This was followed by birds (Table 2; Fig. 6C), amphibians (Table 2; Fig. 6A), and then reptiles (Table 2; Fig. 6B), although amphibians required the least sampling effort for duration in areas of low species richness (Table 2; Fig. 6A). Mammals also required the greatest distance for adequate sampling for all three diversity levels (Table 2; Fig. 7D) followed by birds (Table 2; Fig. 7C), reptiles (Table 2; Fig. 7B), and amphibians (Table 2; Fig. 7A) for all three diversity levels.

**Table 2:** Species richness and sampling frequency over a 40-day period for all four vertebrate taxonomic groups (Amphibia, Reptilia, Aves, and Mammalia) on a 100-km transect in the GMTFCA showing the sampling frequency required for three species diversity categories (high, intermediate, and low) with observed species richness (Mao Tau) and two estimators, Chao 2 and ICE. Adequate sampling was defined as the point which the estimated richness was equal to or less than the richness observed by daily sampling. (Real data are shown in bold and proposed sampling frequency and distance in italics. For example, to sample for mammals in an area of low species richness, we propose sampling for duration of 61 days over a distance of 100 km or 125 kms over a period of 40 days.)

Species diversity category	# of species	Frequency (days)			Distance (km)		
		Estimators		Observed	Estimators		Observed
		Chao 2	ICE	Mao Tau	Chao 2	ICE	Mao Tau
Amphibia							
High	8	*55*	49	40	*100*	107	100
Intermediate	4	*49*	59	40	*100*	116	100
Low	**2**	***3***	**3**	**40**	***4***	**4**	**100**
Reptilia							
High	**27**	***45***	**50**	**40**	***111***	**126**	**100**
Intermediate	13	*45*	49	40	*112*	116	100
Low	7	*38*	47	40	*97*	105	100
Aves							
High	**53**	***59***	**67**	**40**	***145***	**149**	**100**
Intermediate	27	*56*	62	40	*129*	143	100
Low	14	*52*	79	40	*140*	192	100
Mammalia							
High	**24**	***65***	**78**	**40**	***179***	**222**	**100**
Intermediate	12	*58*	92	40	*158*	203	100
Low	6	*61*	61	40	*125*	175	100

## Discussion

### Speed trials

A number of interacting factors will influence the optimum design for a survey of roadkill. These will include the research question being asked, the biodiversity survey that uses roadkill as a tool, and a study of the effect of roadkill on rare species. The size of the target species will influence survey design, and smaller species are likely to require lower sampling speeds. The density of rare species and the propensity of species to use roads will influence how often they are killed and the length of the transect. The length of road that has to be surveyed and the hours of daylight will combine to set a minimum speed.

Recommended speeds vary from slower than 30 km·h^−1^ (e.g., Gomes et al. 2009; Carvalho and Mira 2011; Santos et al. 2011), 45–55 km·h^−1^ (e.g., MacKinnon et al. 2005; Barrientos and Bolonio 2009; Guinard et al. 2012) to greater than 55 km·h^−1^ (e.g., Meunier et al. 2000; Antworth et al. 2005; Barthelmess and Brooks 2010). While detection of small vertebrate prey may be more reliable at slower speeds (Jackson 2003; Langen et al. 2012), MacKinnon et al. (2005) drove at 40–60 km·h^−1^ for detecting snake and turtle roadkill, Sutherland et al. (2010) drove at up to 56 km·h^−1^ for the detection of amphibian roadkill, and Brockie et al. (2009) drove at between 50–100 km·h^−1^ to detect animals of at least “rat size.” However, none of these studies experimentally established the detection rate at the chosen speed (Erritzøe et al. 2003), and based on our results, it is likely that 30 km·h^−1^ was suboptimal and time was wasted. Above 50 km·h^−1^ was also suboptimal and kills were likely missed. Studies of larger species such as mule deer (*Odocoileus hemionus*; Romin and Dalton 1992), raptors (Meunier et al. 2000), and medium-sized mammals (1.0–10.0 kg; Barthelmess and Brooks 2010) have used higher speeds (60–72 km·h^−1^) supporting the contention that species size will influence optimum speed.

Driver safety is an important consideration when determining the most appropriate speed to travel. Driving at a speed of not less than 10–20 km·h^−1^ below the posted speed limit may be recommended for the safety of other drivers and researchers (Clevenger et al. 2003). Our results demonstrate that detection rates decreased significantly at speeds faster than 50 km·h^−1^, and we recommend that, where possible, a maximum speed of 50 km·h^−1^ be driven to obtain reliable data that are cost- and time-effective. Some studies (Slater 2002; Santos et al. 2011) propose conducting on-foot roadkill surveys. However, this is more time-consuming, resulting in shorter overall sampling distances. Therefore, it is recommended that roadkill transects be conducted by vehicle with further study conducted on-foot to look for roadkill that may have gone undetected or to target-specific locations where small-bodied species may occur.

Our study is the first to test for a difference in roadkill detection rate with observer as driver or observer as passenger. Most previous studies do not state if the observer was the driver or the passenger (Case 1978; Serrano et al. 2002; Bullock et al. 2011), and of those that do, the majority had two people in the vehicle with the observer as the passenger) (Clevenger et al. 2003; Russell et al. 2009). In our study, there was no significant difference between detection rates with observer as driver or as passenger, and it will be more cost-effective to have a single person in the vehicle, with the observer as the driver. However, to ensure safety while driving and observing on roads with high traffic volume, a driver and an observer may be considered to ensure the safety of the researcher and other users of the road. In our study, there was a significant difference between detection rates by experienced and inexperienced observers and we recommend that the observer should always be experienced and, for consistency, should be the same person.

Most previous studies (60%) conducted field transects at dawn (e.g., Hels and Buchwald 2001; Ciesiolkiewicz et al. 2006; Barthelmess and Brooks 2010) or shortly after (Meunier et al. 2000; Clevenger et al. 2003; Barthelmess and Brooks 2010), but none tested the effect of time of day on detectability. A range of factors may influence the optimal time to start and end a survey of roadkill. Traffic volumes are lower at dawn due to general working hours (pers.obs.), and this may justify starting roadkill surveys as early in the day as possible. In addition, if surveys start at dawn, there is likely to be less damage to and/or removal of the roadkill carcasses (Hels and Buchwald 2001). With fewer vehicles on the road, it is also likely to be safer for the observers to stop/start during transects (Clevenger et al. 2003). The activity pattern of a target species may influence the timing of the survey. Jackson (2003) surveyed at night to examine the impact of roads on nightjars (Caprimulgidae) which are nocturnal, and Russell et al. (2009) surveyed at dawn and dusk for bats. Although we expected the position of the sun in relation to the direction travelled to significantly influence detectability of roadkill (see Haby 2012; Rossier 2012), this was not the case and there was no significant effect of light conditions. However, the early morning and late afternoon glare from the sun affecting both the researcher and drivers of other vehicles resulted in transects starting at 1.5 h after dawn and ending no later than 1.5 h before dusk.

### Field transects

Previous studies have used a range of durations and sampling frequencies, and consequently, it is difficult to make comparisons between studies (Erritzøe et al. 2003). Of the studies that sampled all vertebrates and were therefore most similar to ours, data were collected daily (Smit and Meijer 1999; Ciesiolkiewicz et al. 2006; Santos et al. 2011), weekly (Taylor and Goldingay 2004; Barthelmess and Brooks 2010; Bager and da Rosa 2011), twice per month (Barrientos and Bolonio 2009; Carvalho and Mira 2011; Quintero-Angel et al. 2012), and monthly (Vestjens 1989; Coelho et al. 2008). In almost all of these studies, the frequency of data collection was not assessed experimentally and no justification was provided. However, Santos et al. (2011) justified daily surveys on the basis of the removal rate of roadkill, even for larger (>10 kg) species. Bager and da Rosa (2011), who sampled weekly over 2 years, stated that weekly sampling was adequate for reptiles and medium-sized mammals but did not attain sampling sufficiency when all vertebrate classes were considered together. To our knowledge, no study has proposed a minimal duration (length of the study), although the daily transects used in our protocol to survey all vertebrate roadkill is supported by Bager and da Rosa (2011) and Santos et al. (2011).

In our study, 65 consecutive days (using the Chao 2 estimator) were adequate to sample all four taxa in areas of high species diversity, with an estimated maximum of 61 days for areas that are of intermediate and low species diversity. Despite birds being the most diverse group, with the greatest number of roadkill recorded, mammals required the greatest sampling effort. This can be correlated with body size, as larger animals often occur at lower densities than smaller animals so, as body size increases, there are simply fewer animals available to be killed (Ford and Fahrig 2007). These estimates can thus be used as a baseline for more species-specific or seasonal-specific roadkill occurrence.

As with sampling frequency, previous studies have used a wide range of sampling distances (e.g., Bager and da Rosa 2011; Carvalho and Mira 2011) making it difficult to compare their results (Erritzøe et al. 2003). The distance used is influenced by a number of factors including the question being asked, the target species, and localized conditions. For example, Loughry and McDonough (1996) sampled a 5-km stretch of road to measure Armadillo (*Darypus novemcinctus*) roadkill and compared this population with a live population at another site, while Gerht (2002) conducted a 41.8-km roadkill survey to obtain mortality indices for raccoon (*Procyon lotor*) populations. This formed part of a larger study monitoring raccoon population demography over an area of 32.39 km^2^.

Our analysis indicated that a sampling distance of 179 km (using the Chao 2 estimator) is required to adequately sample the four vertebrate taxa in areas of high species diversity (with mammals requiring the greatest sampling effort). This distance is slightly reduced for intermediate (158 km) and low (140 km) species richness areas. However, because the goal is a standardized protocol for assessment of roadkill, we recommend a minimum distance of 179 km (where possible, and dependent on the proposed research question) and that future surveys use species accumulation to ensure that an accurate estimate of species richness is obtained.

For data to be comparative (both longitudinally and internationally) in future roadkill detection research and for surveys sampling all vertebrate taxonomic groups, we recommend the identification of a species diversity level (high, intermediate, or low), when vertebrate species are most active and when migratory species were most likely to be present. For individual vertebrate taxonomic groups, we suggest using Table 2 as a guide.

## Conclusion

Our results expand upon components of other wildlife roadkill studies, such as the speed driven (e.g., Barrientos and Bolonio 2009), the number of observers to be used (e.g., Grilo et al. 2009), and the time of day to commence transects (e.g., Barthelmess and Brooks 2010) and create a standardized time- and therefore cost-effective protocol. To our knowledge, no other studies examine all of these variables in detail, although many existing studies provide valuable information on temporal and spatial effects of roadkill (e.g., Clevenger et al. 2003; Conrad and Gipson 2006; Grilo et al. 2009).

It is important that future research becomes more standardized to enable statistical comparisons between different studies and sites, and over time. The conservation implications of our protocol are far-reaching because roads are a necessary component of economic development and yet negatively impact upon biodiversity. In light of expanding road networks, and before it is too late for our wildlife populations, we would recommend that this protocol be the basis of a normalized method to allow comparative studies across the globe and to inform strategies to urgently address this threat**.**

## Supporting Information

**Appendix S1.** A summary of previous roadkill studies (from peer-reviewed published journals) comparing sample methods used to detect roadkill. **Appendix S2.** Supporting references for the summary of 61 previous roadkill studies (from peer-reviewed published journals) comparing sample methods used to detect roadkill. **Appendix S3.** The vertebrate roadkill species detected over a 40-day period on the 100-km section of paved road in the GMTFCA, South Africa.
