# Supplementary material for: Wildlife road traffic accidents: a standardized protocol for counting flattened fauna
Source: Ecol Evol. 2014 Jul 10;4(15):3060–71. doi: 10.1002/ece3.1097 (PMC4161179; doi:10.1002/ece3.1097)
Supplement: Supplementary file 1 — Appendix S1. A summary of previous roadkill studies (from peer-reviewed published journals) comparing sample methods used to detect roadkill. Appendix S2. Supporting references for the summary of 61 previous roadkill studies (from peer-reviewed published journals) comparing sample methods used to detect roadkill. Appendix S3. The vertebrate roadkill species detected over a 40-day period on the 100-km section of paved road in the GMTFCA, South Africa. [file ece30004-3060-sd1.docx]

**Supporting information and appendices**

**Appendix S1** A summary of previous roadkill studies (from peer-reviewed published journals) comparing sample methods used to detect roadkill. (Sampling distance (km) includes either total transect length of length of repeated transects, as stated in the literature).

| **#** | **Author** | **Year** | **Where** | **Species** | **Speed (km h^-1^)** | **# of observers** | **Sampling frequency (when)** | **Sampling frequency (months)** | **Sampling distance (km)** | **Road type** | | **# of transects (repeated)** | **Time of day (start)** | **Other** | **Traffic count** |
| --- | --- | --- | --- | --- | --- | --- | --- | --- | --- | --- | --- | --- | --- | --- | --- |
| **1** | Adams & Geis | 1983 | USA | Small mammals | Walk | 1 | June | 1 | 1.6 | Highways & state roads | | 5 | Any | On-foot surveys on road verges | No |
| **2** | Antworth *et al*. | 2005 | Florida, USA | Vertebrates | 50-80 | No data | 36-hour period | 3 | 34 | Two-lane highway | | 2 | No data | - | No |
| **3** | Bager & da Rosa | 2011 | Southern Brazil | Vertebrates | 50 | No data | Weekly | 36 | 117 | Federal highway | | 1 | No data | - | No |
| **4** | Barrientos & Bolonio | 2009 | Central Spain | Polecat (*Mustela putorius* L.) | 40-50 | 2 | Bi-monthly | 23 | 246 | No data | | 2 | No data | Additional random on- foot surveys (road verges) | No |
| **5** | Barthelmess & Brooks | 2010 | New York, USA | Mammals | 72 | 2 | Weekly | 14 | 206.3 | No data | | 3 | 06:30 and 07:00 | - | No |
| **6** | Bright *et al.* | 2005 | UK | Mammals | No data | No data | Over 3 months | monthly | >32 | All roads excluding motorways/dual carriageways | | Numerous | No data | Data taken from volunteers across the UK | Yes |
| **7** | Brockie *et al.* | 2009 | North Island, New Zealand | Mammals & birds | 50-100 | 2 | February (over 3 years) | 3 | 1660 | No data | | 16 | Daylight hours | - | No |
| **8** | Bullock *et al.* | 2011 | RSA | Mammals & birds | 100 | No data | 12 surveys | 9 | 261 | National road & gravel | | 1 | No data | - | No |
| **9** | Caro *et al.* | 2000 | California, USA | Medium-sized mammals | No data | No data | 10 – 30 times a month | 25 | 14.2, 12,9 12.8 | Two-lane paved roads | | 3 | Daylight | Some transects were driven twice a day in different directions | No |
| **10** | Case | 1978 | Nebraska, USA | Birds and mammals | No data | No data | No data | 84 | 732 | Highway | | 1 | No data | Data reported by service crew | Yes |
| **11** | Carvalho & Mira | 2011 | Portugal | Vertebrates | 20 | No data | Bi-monthly | 24 | 26 | National road | | 1 | No data | - | No |
| **12** | Ciesiolkiewicz *et al.* | 2006 | Poland | Snakes | N/A | 1 | Daily for 3 months & then twice a week for remaining | 11 | 1.8 | Tarmac road | | 1 | Between 06:00 & 10:00 | Walking survey (both sides of the road) | No |
| 13 | Clevenger *et al.* | 2003 | Alberta, Canada | Small vertebrates | 10-20 below posted speed limit | 2 | Daily | 36 | 105.6 & 142.5 | No data | | 2 (alternated each day) | 1 hour after sunrise | - | Yes |
| 14 | Coelho *et al.* | 2008 | Brazil | Vertebrates | 40-60 | 2 | Monthly | 12 | 195 | National highway | | 2 | No data | No count for amphibians due to size | Yes |
| 15 | Conrad & Gibson | 2006 | Kansas, USA | Mammals | 55-65 | No data | Weekly (over 2 years) | 7 | 40 | State highway | | 2 | No data | - | Yes |
| **#** | **Author** | **Year** | **Where** | **Species** | **Speed (km h^-1^)** | **# of observers** | **Sampling frequency (when)** | **Sampling frequency (months)** | **Sampling distance (km)** | **Road type** | **# of transects (repeated)** | | **Time of day (start)** | **Other** | **Traffic count** |
| 16 | Da Rosa & Bager | 2012 | Brazil | Birds | 50 | 2 | 95 monitorings | 27 | 117 | Highway | 2 | | 07:00 | No weekends and no days when there was rain | No |
| 17 | Dean & Milton | 2003 | Nama-Karoo, RSA | Raptors | 100 | 2 | 6 - 26 surveys per month | 162 | 90, 012 | No data | 1 | | No data | Data recorded on a tape recorder | No |
| 18 | Dickerson | 1939 | USA (14 states) | Vertebrates | No data | 1 & 2 | 62 days | 36 | >75, 000 | National roads | Various | | No data | - | No |
| 19 | Dreyer | 1935 | USA (3 states) | Vertebrates | No data | No data | 9 days | N/A | 1, 500 | National road | 2 | | No data | -- | No |
| 20 | Gerht | 2002 | Illinois, USA | Raccoon (*Procyon lotor*) | 16-24 | 1 & 2 | Bi-weekly | 24 | 41.8 | No data | 3 | | No data | - | Yes |
| 21 | Gomes *et al.* | 2009 | Portugal | Owls (Strigiformes) | 30 | 1 | Bi-monthly | 24 | 622 | No data | 14 | | No data | - | No |
| 22 | Grilo *et al.* | 2009 | Portugal | Carnivores | 30 | 1 | Bi-monthly | 24 | 574 | Highway and national road | 2 | | No data | Both directions driven | Yes |
| 23 | Guinard *et al.* | 2012 | France | Birds | 40-50 | 2 | 2.5 days per season totalling 10 a year | 24 | 166 | Highway | 1 | | 2 counts per day ( no count at night) | Random foot surveys conducted over 10 km | Yes |
| 24 | Gunson *et al.* | 2003 | Canada | Large animals | No data | No data | No data | 12 | Part of study area | Highway | Numerous | | No data | Data taken from insurance company reports of animal/vehicle collisions | Yes |
| 25 | Haikonen & Summala | 2001 | Finland | Moose *(Alces alces)* & White-tailed deer (*Odocoileus virginianus*) | No data | No data | Over 9 years | No data | Part of study area | No data | Numerous | | No data | Data taken from crash statistics | Yes |

| 26 | Hegel *et al.* | 2012 | Brazil | Mammals | 60 | No data | No data | 24 | 3720 | Highway | 1 | No data | - | No |
| --- | --- | --- | --- | --- | --- | --- | --- | --- | --- | --- | --- | --- | --- | --- |
| **27** | Hell *et al.* | 2004 | Slovak | Vertebrates | No data | No data | Weekly | 28 | 32 | Highway | 4 | No data | Monitored by bike / car / on foot | - |
| **28** | Hels & Buchwald | 2000 | Denmark | Amphibians | No data | No data | Over 3 years | 7 | 0.6 | No data | 1 | Dawn | During breeding seasons | - |
| **29** | Jackson | 2003 | Zimbabwe | Nightjars (Caprimulgidae) | 15-25 | No data | Weekly | 12 | 32 | No data | 1 | Night | Both directions driven | No |
| **30** | Joyce & Mahoney | 2001 | Canada | Moose *(Alces alces)* | No data | No data | Over 6 years | No data | Part of study area | All road types | Numerous | No data | Data taken from crash statistics | Yes |
| **31** | Kleist *et al.* | 2007 | North Carolina, USA | White-tailed deer (*Odocoileus virginianus*) | No data | No data | Weekly | 17 | 1.8 | Highway | 1 | No data | Video surveillance also used | No |
|  |  |  |  |  |  |  |  |  |  |  |  |  |  |  |
| **#** | **Author** | **Year** | **Where** | **Species** | **Speed (km h^-1^)** | **# of observers** | **Sampling frequency (when)** | **Sampling frequency (months)** | **Sampling distance (km)** | **Road type** | **# of transects** | **Time of day (start)** | **Other** | **Traffic count** |
| **32** | Kolowski & Nielson | 2008 | Illinois, USA | Bobcat *(Lynx rufus)* | No data | No data | Over 12 years | No data | Part of study area | No data | No data | No data | Opportunistically collected | Yes |
| **33** | Langen *et al.* | 2012 | New York State, USA | Freshwater turtles | 32-46 | 2 | Weekly (over 2 years) | 12 | 160 | Highways | 1 | 06:30-12:00 | Direction alternated | Yes |
|  |  |  |  |  |  |  |  |  |  |  |  |  |  |  |
| **34** | Loughry & McDonough | 1996 | Florida, USA | Armadillo (*Darypus novemcinctus)* | No data | No data | Over 3 years | 3 (August) | 5 | Highway | 1 | No data | - | No |
| **35** | McDonald | 2012 | Australia | Snakes | 40-60 | No data | 77 occasions | 12 | 77 | Sealed road | 1 | 1 hour after sunset | Both directions driven | No |
| **36** | MacKinnon *et al.* | 2005 | Ontario, Canada | Reptiles | 40-60 | No data | April – October (over 2 years) | 14 | 12.2 | No data | 1 | Daylight hours | - | No |
| **37** | Malo *et al.* | 2004 | Spain | Vertebrates | No data | No data | Over 13 years | No data | 3253 | Motorway | No data | No data | Data taken from database on traffic collisions | Yes |
| **38** | Markolt *et al.* | 2012 | Hungary | Large mammals | No data | No data | Over 7 years | No data | 223 | Highway | 1 | No data | Data taken from State motorway management Company database | No |
| **39** | Mkanda & Chansa | 2010 | Zambia | Vertebrates | No data | No data | No data | 29 | ~80 | Highway | No data | No data | - | Yes |
| **40** | Meunier *et al*. | 2000 | France | Raptors | 60-70 | No data | 7 periods, 2 months apart | 12 | 2772 | Motorway & secondary roads | No data | 3 different hours of day | Alternated driving direction | Yes |
| **41** | Mohammadi *et al.* | 2011 | Iran | Long-eared hedgehog *(Hemiechinus auritus)* | No data | No data | No data | 1 day | No data | No data | No data | No data | - | No |
| **42** | Neumann *et al.* | 2012 | Sweden | Moose *(Alces alces)* | No data | No data | No data | 24 | Part of study area | No data | No data | No data | Data taken from police reports | Yes |
|  |  |  |  |  |  |  |  |  |  |  |  |  |  |  |
|  |  |  |  |  |  |  |  |  |  |  |  |  |  |  |
| **43** | Orlowski | 2008 | Poland | Birds | 20-50 | No data | Bi-weekly | 26 | 48.8 | No data | 15 | Afternoon | - | Yes |
| **44** | Quintero-Angel *et al.* | 2012 | Columbia | Snakes | Walk | 2 | Bi-monthly | 5 | No data | No data | No data | No data | - | Yes |
| **45** | Ramp *et al.* | 2005 | NSW Australia | Mammals | 60 | 1 | 5 days a week | 168 | 40 | No data | 1 | Twice a day | - | Yes |
| **46** | Rodda | 1990 | Venezuela | Iguanas | No data | No data | No data | 14 | ~1000 (per month) | Highway | No data | No data | - | No |
| **47** | Romin & Dalton | 1992 | Utah, USA | Mule deer (*Odocoileus hemionus*) | 65 | No data | January & February | 2 | 9.7 | Dirt road | 1 | No data | - | No |
| **48** | Russell *et al.* | 2009 | Pennsylvania, USA | Bats | Walk | 2 | Arbitrary days | 3 | 5 | Highway | 1 | Dusk & dawn | - | No |
| **49** | Saeki & MacDonald | 2004 | Japan | Raccoon dog *(Nyctereutus procyonoides viverrinus)* | No data | No data | Several times a day | 36 | 627.3 | National expressways | 44 | No data | Data collated from Japan Highway Corporation during routine road checks | Yes |
| **#** | **Author** | **Year** | **Where** | **Species** | **Speed (km h^-1^)** | **# of observers** | **Sampling frequency (when)** | **Sampling frequency (months)** | **Sampling distance (km)** | **Road type** | **# of transects** | **Time of day (start)** | **Other** | **Traffic count** |
| **50** | Santos *et al.* | 2011 | Review | Vertebrates | No data | No data | Daily | No data | No data | No data | 4 | No data | - | Yes |
| **51** | Serrano *et al.* | 2002 | Spain | Large/medium sized mammals | No data | No data | Weekly | 24 | 55 | Freeways | 2 | No data | - | Yes |
| **52** | Siegfried | 1966 | South Africa | Birds | Slowly | 2 | Twice daily | 24 | 14.5 | Paved | 1 | 08:00-17:00 | - | No |
| **53** | Seiler | 2005 | Sweden | Moose *(Alces alces)* | No data | No data | Daily | 108 | Part of study area | No data | Numerous | No data | Data taken from police reports | Yes |
| **54** | Seshadri *et al.* | 2009 | India | Amphibians | No data | No data | Daily | 4 days | 25 | No data | 1 | 06:30-08:30 | - | - |
| **55** | Slater | 2002 | Wales, UK | Mammals | No data | 2 | Bi-weekly | 12 | 68 | No data | 1 | Dawn onwards | Four 2 km lengths walked by other staff | No |
| **56** | Smit & Meijer | 1999 | Holland | Vertebrates | No data | No data | ~Daily | 96 | No data | National highways | No data | No data | Conducted by road inspectors | No |
| **57** | Smith-Patten & Patten | 2008 | Kansas, USA | Mammals (medium sized) | No data | No data | Bi-monthly | 36 | No data | No data | No data | Daylight hours | Surveys were opportunistic. | No |
| **58** | Snow *et al.* | 2011 | California, USA | San Clement Island Fox *(Urocyon littoralis clementae)* | 56 | No data | 4-7 times per week | 29 | 32.2 | Paved & gravel | 6 | No data | - | Yes |
| **59** | Stoner | 1925 | Iowa, USA | Vertebrates | ~40 | 2 | 4 days | ~2 | 508.6 | Paved, gravel & dirt | Many | No data | - | No |
| **60** | Sutherland *et al.* | 2010 | North Carolina, USA | Amphibians | 48–56 | No data | Daily over 2 months for 2 years | 2 | 144 | No data | 2 | Night | - | Yes |
| **61** | Taylor & Goldingay | 2004 | Australia | Vertebrates | 70-80 | No data | 20 weekly | 12 | 100.3 | Major highway | 3 | No data | - | Yes |

**Appendix S2** Supporting references for the summary of 61 previous roadkill studies (from peer-reviewed published journals) comparing sample methods used to detect roadkill (not all cited in this manuscript).

**References**

1. Adams, L.W. & Geis, A.D. (1983) Effects of roads on small mammals. *Journal of Applied Ecology*, **20**, 403-415.
2. Antworth, R.L., Pike, D.A. & Stevens, E.E. (2005) Hit and run: effects of scavenging on estimates of roadkill vertebrates. *Southeastern Naturalist*, **4**, 647-656.
3. Bager, A. & da Rosa, C.A. (2011) Influence of sampling effort on the estimated richness of road-killed vertebrate wildlife. *Environmental Management*, **47**, 851-858.
4. Barrientos, R. & Bolonio, L. (2009) The presence of rabbits adjacent to road increases polecat road mortality. Biodiversity Conservation, 18, 405-418.
5. Barthelmess, E.L. & Brooks, M.S. (2010) The influence of body-size and diet on road-kill trends in mammals. *Biodiversity Conservation,* **19**, 1611-1629.
6. Bright, P., George, L. & Bamlforth, Z. (2005) Mammals on roads. Development and testing the use of road counts to monitor abundance. A report to People’s Trust for Endangered Species Joint Nature Conservation Committee, (Draft v 9).
7. Brockie, R.E., Sadleir, R.M.F.S. & Linklater, W.L. (2009) Long-term wildlife road-kill counts in New Zealand. *New Zealand Journal of Ecology*, **36**, pp. 123-134.
8. Bullock, K.L., Malan, G. & Pretorius, M.D. (2011) Mammal and bird road mortalities on the Uppington to Twee Rivieren main road in the Southern Kalahari, South Africa. *African Zoology*, **46**, 60-71.
9. Caro, T.M., Shargel, J.A. & Stoner, C.J. (2000) Frequency of medium-sized mammal road kills in an agricultural landscape in California. *American Midland Naturalist,* **144**, 362-369.
10. Carvalho, F. & Mira, A. (2011) Comparing annual vertebrate road kills over two time periods, 9 years apart: a case study in Mediterranean farmland. *European Journal of Wildlife Research*, **57**, 157-174.
11. Case, R.M. (1978) Highway road-killed animals: a data source for biologists. *Wildlife Society Bulletin*, **6,** 8-13.
12. Ciesiolkiewicz, J., Orlowski, G. & Elzanowski, A. (2006) High juvenile mortality of grass snakes *Natrix natrix* (L.) on a suburban road. *Polish Journal of Ecology*, **54,** 465-472.
13. Clevenger, A.P., Chruszcz, B. & Gunson, K.E. (2003) Spatial patterns and factors influencing small vertebrate fauna road-kill aggregations. *Biological Conservation*, **109**, 15-26.
14. Coelho, I.P. Kindel, A. & Coelho, A.V.P. (2008) Roadkills of vertebrate species on- two highways through the Atlantic Forest Biosphere Reserve, Southern Brazil. *European Journal of Wildlife Research*, **54,** 689-699.
15. Conrad, J.M. & Gipson, P.S. (2006) Spatial and seasonal variation in wildlife-vehicle collisions. *The Prairie Naturalist,* **38**, issue.
16. Da Rosa, C.A. & Bager, A. (2012) Seasonality and habitat types affect roadkill of neotropical birds. *Journal of Environmental Management*, **97**, 1-5.
17. Dean, W.R.J. & Milton, S.J. (2009) The importance of roads and road verges for raptors and crows in the succulent and Nama-Karoo, South Africa. *OSTRICH, Journal of African Ornithology*, **74**, issue. 3, 181-186.
18. Dickerson, L.M. (1939) The problem of wildlife destruction by automobile traffic. *The Journal of Wildlife Management,* **3**, No. 2, 104-116.
19. Dreyer, W.A. (1935) The question of wildlife destruction by the automobile. *Science* **82**, 439–440.
20. Gerht, S.D. (2002) Evaluation of spotlight and road-kill surveys as indicators of local raccoon abundance. *Wildlife Society Bulletin,* **30**, No. 2, 449-456.
21. Gomes, L.,Grilo, C., Silva, C.& Mira, A. (2009) Identification methods and deterministic factors of owl roadkill hotspot locations in Mediterranean landscapes. *Ecological Research*, **24**, 355-370.
22. Grilo, C., Bissonette, J.A. & Santos-Reis, M. (2009) Spatial-temporal patterns in Mediterranean carnivore road casualties: consequences for mitigation. *Biological Conservation*, **142**, 301-313.
23. Guinard, E., Julliard, R. & Barbruad, C. (2012) Motorways and bird traffic casualties: carcasses surveys and scavenging bias. *Biological Conservation*, **147**, 40-51.
24. Gunson, K.E., Chruszcz, B. & Clevenger, A.P. (2003) Large animal-vehicle collisions in the Central Canadian Rocky Mountains: patterns and characteristics. Road Ecology Center, John Muir Institute of the Environment, UC Davis. Proceedings of the 2003 International Conference on Ecology and Transportation and the Environment, North Carolina State University, Raleigh, NC: pp. 355-366.
25. Haikonen, H. & Summala, H. (2001) Deer-vehicle crashes: extensive peak 1 hour after sunset. *Journal of Preventative Medicine*, **21**, 209-213.
26. Hegel, C.G.Z., Consalter, G.C. & Zanella, N. (2012) Mamiferos silvestres astropelados na rodovia RS-135, norte do Estado do Rio Grande do Sul. *Revista Biotemas*, **25**, 165-170.
27. Hell, P., Plavy, R., Slamečka, J. & Gašparik, J. (2005) Losses of mammals (Mammalia) and birds (Aves) on roads in the Slovak part of the Danube Basin. *European Journal of Wildlife Research*, **51**, 35-40.
28. Hels, T. & Buchwald, E. (2001) The effect of road kills on amphibian populations. *Biological Conservation,* **99**, 331-340.
29. Jackson, H.D. (2003) A field survey to investigate why nightjars frequent roads at night. *OSTRICH, Journal of African Ornithology*, **74**, issue. 1, 97-101.
30. Joyce, T.L. & Mahoney, S.P. (2001) Spatial and temporal distributions of moose-vehicle collisions in Newfoundland. *Wildlife Society Bulletin*, **29,** 281-291.
31. Kleist, A.M., Lancia, R.A. & Doeer, R.A. (2007) using video surveillance to estimate wildlife use of a highway underpass. *The Journal of Wildlife Management,* **71**, 2792-2800
32. Kolowski, J. M. & Nielsen, C. K. (2008) Using Penrose distance to identify potential risk of wildlife–vehicle collisions. *Biological Conservation*, **141** (4): 1119-1128.
33. Langen, T.A., Gunson, K.E., Scheiner, C.A. & Boulerice, J.T. (2012) Road mortality in freshwater turtles: identifying causes of spatial patterns to optimize road planning and mitigation. *Biodiversity and Conservation,* **21**, 3017-3034.
34. Loughry, W.J. & McDonough, C.M. (1996) Are road kills valid indicators of armadillo population structure? *American Midland Naturalist*, **135**, 53-59.
35. MacKinnon, C.A., Moore, L.A. & Brooks, R.J. (2005) Why Did the reptile cross the road? landscape factors associated with road mortality of snakes and turtles in the South Eastern Georgian Bay area. Parks Research Forum of Ontario.*2005 PRFO Proceedings.*
36. Malo, J.E., Suarez, F. & Diez, A. (2004) Can we mitigate animal-vehicle accidents using predictive models? *Journal of Applied Ecology*, **41,** 701-710.
37. Markolt, F., Szemethy, L., Lehoczki, R. & Heltai, M. (2012) Spatial and temporal evaluation of wildlife-vehicle collisions along the M3 Highway in Hungary. *North-Western Journal of Zoology*, **8** (2): on-first.
38. McDonald, P.J. (2012) Snakes on roads: an arid Australian perspective. *Journal of Arid Environments,* **79**, 116-119.
39. Meunier, F.D., Verheyden, C. & Jouventin, P. (2000) Use of roadsides by diurnal raptors in agricultural landscapes. *Biological Conservation*, **92**, 291-298.
40. Mkanda, F.X. & Chansa, W. (2010) Changes in temporal and spatial pattern of road kills along the Lusaka-Mongu (M9) highway, Kafue National Park, Zambia. *South African Journal of Wildlife Research*, **41**, 68-78.
41. Mohammadi, S., Farahi, M., Ganjali, M. & Kookhan, S. (2011) Roadkill mortality of the long-eared hedgehog *Hemiechinus auritus* from Zabol, SE Iran. *European Journal of Biological Sciences*, **3,** 99-101.
42. Neumann, W., Ericsson, G., Dettki, H., Bunnefeld, N., Keuler, N.S., Helmers, D.P. & Radeloff, V.C. (2012) Difference in spatiotemporal patterns of wildlife road-crossings and wildlife-vehicle collisions. *Biological Conservation*, **145**, 70-78.
43. Orlowski, G. (2008) Roadside hedgerows and trees as factors increasing road mortality of birds: Implications for management of roadside vegetation in rural landscapes. *Landscape and Urban Planning*, **86,** 153-161.
44. Quintero-Angel, A., Osorio-Dominguez, D., Vargas-Salinas, F. & Saavedra-Rodriguez, C.A. (2012) Roadkill rate of snakes in a disturbed landscape of Central Andes of Columbia. *Herpetology Notes*, **5**, 99-105.
45. Ramp, D., Caldwell, J., Edwards, K.A., Warton, D. & Croft, D.B. (2005) Modelling of wildlife fatality hotspots along the Snowy Mountain Highway in New South Wales, Australia. *Biological Conservation*, **126**, 474-490.
46. Rodda, G.H. (1990) Highway madness revisited: roadkilled *Iguana iguana* in the Llanos of Venezuela. *Society for the Study of Amphibians and Reptiles*, **24,** 209-211.
47. Romin, L.A. & Dalton, L.B. (1992) Lack of response by mule deer to wildlife warning whistles. *Wildlife Society Bulletin*, **20**, No. 4, 382-384.
48. Russell, A.L., Butchkoski, C.M., Saidak, L. & McCracken, G.F. (2009) Road-killed bats, highway design, and the commuting ecology of bats. *Endangered Species Research*, **8**, 49-60.
49. Saeki, M. & MacDonald, D.W. (2004) The effects of traffic on the raccoon dog (*Nyctereutes procyonoides viverrinus*) and other mammals in Japan. *Biological Conservation*, 118: 559-571.
50. Santos, S.M. Carvalho, F. & Mira, A. (2011) How long do the dead survive on the road? Carcass persistence, probability and implications for road-kill monitoring surveys. *PLoS ONE*, **6**, issue. 9.
51. Seiler, A. (2005) Predicting locations of moose-vehicle collisions in Sweden. *Journal of Applied Ecology*, **42,** 371-382.
52. Serrano, M., Sanz, L., Puig, J., & Pons, J. (2002) Landscape fragmentation caused by the transport network in Navarra (Spain): two-scale analysis and landscape integration assessment. *Landscape and Urban Planning*, **58** (2): 113-123.
53. Seshadri, K.S., Yadav, A. & Gururaja, K.V. (2009) Road kills of amphibians in different land use areas from Sharavathi river basin, central Western Ghats. *Journal of Threatened Taxa,* **1,** 549-552.
54. Siegfried, W.R. (1965) A survey of wildlife mortality on roads in the Cape Province. *Cape Department of Nature Conservation Investigational Report* No. 6, Cape Town, South Africa.
55. Slater, E.M. (2002) An assessment of wildlife road casualties – the potential discrepancy between numbers counted and numbers killed. *Web Ecology*, **3**, 33-42.
56. Smit, G.F.J. & Meijer, A.J.M. (1999) Experience with the counting of animal road casualties. *Lutra*, **42,** 25-36.
57. Smith-Patten, B.D. Patten, M.A. (2008) Diversity, seasonality and context of mammalian roadkills in the Southern Georgia Plains. *Environmental Management,* **41**, 844-852
58. Snow, N.P., Andelt, W.F. & Gould, N.P. (2011) Characteristics of road-kill locations of San Clemente Island foxes. *Wildlife Society Bulletin*, **35,** 32-39.
59. Stoner, D. (1925) The toll of the automobile. *Science, New Series*, **61**, No. 1568, 56-57.
60. Sutherland, R.W., Dunning, P.R. & Baker, W.M. (2010) Amphibian encounter rates on roads with different amounts of traffic and urbanization. *Conservation Biology,* **24,** issue. 6, 1626-1635.
61. Taylor, B.D. & Goldingay, R.L. (2004) Wildlife roadkills on three major roads in north-eastern New South Wales. *Wildlife Research,* **31,** 83-91.

.

Appendix S3 The vertebrate roadkill species detected over a 40-day period on the 100 km section of paved road in the GMTFCA, South Africa. (Activity: C = Crepuscular, D = Diurnal, N = Nocturnal, B = Both diurnal and nocturnal; Branch 1998, Hockey *et al*. 2005, Skinner & Chimimba 2005, Carruthers & du Preez 2011). The top three species with highest roadkill numbers for three taxonomic groups (Reptilia, Aves and Mammalia) are highlighted in bold. The Amphibia top roadkill species only is highlighted.

| **Class** | **No.** | **Order** | **Family** | **Scientific name** | **Common Name** | **Activity** | **# of roadkill detected over a 40-day period** |
| --- | --- | --- | --- | --- | --- | --- | --- |
| **Amphibia** | 1 | Anura | Breviciptidae | *Breviceps adspersus* | Bushveld Rain Frog | N | 1 |
|  | **2** | **Anura** | **Bufonidae** | ***Amietophrynus garmani*** | **Eastern Olive Toad** | **N** | **4** |
|  |  |  |  |  |  |  |  |
| **Reptilia** | 1 | Testudines | Testudinidae | *Geochelone pardalis* | Leopard Tortoise | D | 2 |
|  | 2 | Testudines | Pelomedusidae | *Pelusios sinuatus* | Serrated Hinged Terrapin | D | 2 |
|  | 3 | Squamata | Boidae | *Python natalensis* | Southern African Python | D | 3 |
|  | 4 | Squamata | Atractaspididae | *Atractaspis bibronii* | Bibron's Burrowing Asp | N | 6 |
|  | 5 | Squamata | Atractaspididae | *Atractaspis duerdeni* | Duerden's Burrowing Asp | N | 4 |
|  | 6 | Squamata | Atractaspididae | *Xenocalamus transvaalensis* | Transvaal Quill-Snouted Snake | D | 1 |
|  | 7 | Squamata | Colubridae | *Dasypeltis scabra* | Rhombic Egg Eater | N | 2 |
|  | 8 | Squamata | Colubridae | *Dispholidus typus* | Boomslang | D | 3 |
|  | **9** | **Squamata** | **Colubridae** | ***Lamprophis fuliginosus*** | **Brown House Snake** | **N** | **13** |
|  | 10 | Squamata | Colubridae | *Mehelya nyassae* | Black File Snake | N | 1 |
|  | 11 | Squamata | Colubridae | *Philothamnus semivariegatus* | Spotted Bush Snake | D | 1 |
|  | 12 | Squamata | Colubridae | *Prosymna bivittata* | Two-striped Shovel Snout | N | 1 |
|  | 13 | Squamata | Colubridae | *Prosymna sundevalli* | Sundevall's Shovel-Snout | N | 2 |
|  | 14 | Squamata | Colubridae | *Psammophis mossambicus* | Olive Grass Snake | D | 1 |
|  | 15 | Squamata | Colubridae | *Psammophis subtaeniatus* | Stripe-bellied Sand Snake | D | 2 |
|  | 16 | Squamata | Colubridae | *Rhamphiophis rostratus* | Rufous Beaked Snake | D | 2 |
|  | 17 | Squamata | Colubridae | *Telescopus semiannulatus* | Eastern Tiger Snake | N | 1 |
|  | 18 | Squamata | Elapidae | *Dendroaspis polyelpis* | Black Mamba | D | 2 |
|  | 19 | Squamata | Elapidae | *Elapsoidea boulengeri* | Boulenger's Garter Snake | N | 1 |
|  | **20** | **Squamata** | **Elapidae** | ***Naja mossambica*** | **Mozambique Spitting Cobra** | **N** | **15** |
|  | 21 | Squamata | Viperidae | *Bitis arientans arientans* | Puff Adder | C | 3 |
|  | 22 | Squamata | Viperidae | *Bitus caudalis* | Horned Adder | C | 6 |
|  | 23 | Squamata | Viperidae | *Causus rhombeatus* | Rhombic Night Adder | N | 5 |
|  | 24 | Squamata | Gerrhosauridae | *Gerrhosaurus nigrolineatus* | Black-lined Plated Lizard | D | 9 |
|  | 25 | Squamata | Varanidae | *Varanus albigularia albigularis* | Rock Monitor | D | 3 |
|  | 26 | Squamata | Agamidae | *Agama armata* | Peter's Ground Agama | D | 1 |
|  | **27** | **Squamata** | **Chamaeleonidae** | ***Chamaeleo dilepsis*** | **Flap-neck Chameleon** | **D** | **41** |
| **Class** | **No.** | **Order** | **Family** | **Scientific name** | **Common Name** | **Activity** | **# of roadkill detected over a 40-day period** |
| **Aves** | 1 | Galliformes | Phasianidae | *Dendroperdix sephaena* | Crested Francolin | D | 3 |
|  | 2 | Galliformes | Phasianidae | *Pternisris natalensis* | Natal Spurfowl | D | 5 |
|  | 3 | Galliformes | Phasianidae | *Coturnix coturnix* | Common Quail | D | 2 |
|  | **4** | **Galliformes** | **Numididae** | ***Numida meleagris*** | **Helmeted Guineafowl** | **C** | **44** |
|  | 5 | Bucertiformes | Bucerotidae | *Tockus erythrohynchus* | Red-billed Hornbill | D | 2 |
|  | 6 | Bucertiformes | Bucerotidae | *Tockus leucomelas* | Southern Yellow-billed Hornbill | D | 2 |
|  | 7 | Coraciiformes | Coraciidae | *Coracias garrulus* | European Roller | D | 3 |
|  | 8 | Coraciiformes | Coraciidae | *Coracias caudatus* | Lilac-breasted Roller | D | 4 |
|  | 9 | Coraciiformes | Meropidae | *Merops pusillus* | Little Bee-Eater | D | 1 |
|  | 10 | Coraciiformes | Meropidae | *Merops nubicoides* | Southern Carmine Bee-eater | D | 7 |
|  | 11 | Culcliformes | Centropodidae | *Centropus burchelii* | Burchell's Coucal | D | 1 |
|  | 12 | Strigiformes | Tytonidae | *Tyto alba* | Barn Owl | N | 2 |
|  | 13 | Strigiformes | Strigidae | *Bubo africanus* | Spotted Eagle Owl | N | 6 |
|  | 14 | Strigiformes | Caprimulgidae | *Caprimulgus pectoralis* | Fiery-necked Nightjar | C | 5 |
|  | 15 | Strigiformes | Caprimulgidae | *Caprimulgus tristigma* | Freckled Nightjar | N | 4 |
|  | 16 | Strigiformes | Caprimulgidae | *Caprimulgus fossii* | Square-tailed Nightjar | N | 4 |
|  | **17** | **Strigiformes** | **Caprimulgidae** | ***Caprimulgus rufigena*** | **Rufous-cheeked Nightjar** | **N** | **17** |
|  | 18 | Strigiformes | Caprimulgidae | *Caprimulgus europaeus* | European Nightjar | N | 8 |
|  | 19 | Strigiformes | Caprimulgidae | *Macrodipteryx vexillarius* | Pennant-winged Nightjar | N | 4 |
|  | 20 | Columbiformes | Columbidae | *Streptopelia senegalensis* | Laughing Dove | D | 2 |
|  | 21 | Columbiformes | Columbidae | *Streptopelia decipiens* | Red-eyed Dove | D | 1 |
|  | 22 | Columbiformes | Columbidae | *Oena capensis* | Namaqua Dove | D | 1 |
|  | 23 | Gruiformes | Otididae | *Lophotis ruficrista* | Red-crested Korhaan | D | 3 |
|  | 24 | Charadriiformes | Burhinidae | *Burhinus capensis* | Spotted Thick Knee | N | 5 |
|  | 25 | Charadriiformes | Glareolidae | *Rhinoptilus chalcopterus* | Bronze-Winged Courser | N | 2 |
|  | 26 | Falconiformes | Accipitridae | *Accipiter tachiro* | African Goshawk | D | 1 |
|  | 27 | Falconiformes | Accipitridae | *Buteo vulpinus* | Steppe Buzzard | D | 1 |
|  | 28 | Passeriformes | Malaconotidae | *Tchagra australis* | Brown-crowned Tchagra | D | 2 |
|  | 29 | Passeriformes | Malaconotidae | *Prionops plumatus* | White-crested Helmet Shrike | D | 2 |
|  | 30 | Passeriformes | Corvidae | *Corvus albus* | Pied Crow | D | 1 |
|  | 31 | Passeriformes | Laniidae | *Lanius collurio* | Red-backed Shrike | D | 3 |
|  | 32 | Passeriformes | Laniidae | *Eurocephalus anguitimens* | Southern White-crowned Shrike | D | 3 |
|  | 33 | Passeriformes | Paridae | *Anthoscopus caroli* | Grey Penduline Tit | D | 1 |
|  |  |  |  |  |  |  |  |
| **Class** | **No.** | **Order** | **Family** | **Scientific name** | **Common Name** | **Activity** | **# of roadkill detected over a 40-day period** |
| **Aves** | 34 | Passeriformes | Hirundinidae | *Hirundo rustica* | Barn Swallow | D | 9 |
|  | 35 | Passeriformes | Sylviidae | *Eromomela icteropygialis* | Yellow-bellied Eremomela | D | 1 |
|  | 36 | Passeriformes | Cisticolidae | *Cisticola aridulus* | Desert Cisticola | D | 1 |
|  | 37 | Passeriformes | Cisticolidae | *Prinia flavicans* | Black-chested Prinia | D | 1 |
|  | 38 | Passeriformes | Cisticolidae | *Heliolais erythropterus* | Red-winged Warbler | D | 1 |
|  | 39 | Passeriformes | Alaudidae | *Mirafra sabota* | Sabota Lark | D | 7 |
|  | 40 | Passeriformes | Muscicapidae | *Melaenornis pammelaina* | Southern Black Flycatcher | D | 1 |
|  | 41 | Passeriformes | Muscicapidae | *Cercotrichas leucophrys* | White-browed Scrub Robin | D | 1 |
|  | 42 | Passeriformes | Ploceidae | *Plocepasser mahali* | White-browed Sparrow-Weaver | D | 4 |
|  | 43 | Passeriformes | Ploceidae | *Anaplectes melanotis* | Red-headed Weaver | D | 1 |
|  | 44 | Passeriformes | Ploceidae | *Quelea quelea* | Red-billed Quelea | D | 1 |
|  | 45 | Passeriformes | Estrildidae | *Amadina fasciata* | Cut-throat Finch | D | 1 |
|  | 46 | Passeriformes | Estrildidae | *Granatina grantina* | Violet-eared Waxbill | D | 1 |
|  | 47 | Passeriformes | Estrildidae | *Uraeginthus angolensis* | Blue waxbill | D | 1 |
|  | 48 | Passeriformes | Estrildidae | *Pytilia melba* | Green-winged Pytilia | D | 1 |
|  | 49 | Passeriformes | Passeridae | *Petronia superciliaris* | Yellow-throated Petronia | D | 2 |
|  | 50 | Passeriformes | Fringillidae | *Crithagra mozambica* | Yellow-fronted Canary | D | 1 |
|  | 51 | Passeriformes | Fringillidae | *Crithagra Flaviventris* | Yellow Canary | D | 1 |
|  | **52** | **Passeriformes** | **Fringillidae** | ***Emberiza tahapisi*** | **Cinnamon-breasted Bunting** | **D** | **13** |
|  | 53 | Passeriformes | Fringillidae | *Emberiza capensis* | Golden-breasted Bunting | D | 5 |
|  |  |  |  |  |  |  |  |
| **Mammalia** | **1** | **Lagomopha** | **Leporidae** | ***Lepus saxatilis*** | **Scrub hare** | **N** | **26** |
|  | 2 | Rodentia | Bathyergidae | *Cryptomys hottentotus* | African mole-rat | N | 1 |
|  | 3 | Rodentia | Hystricidae | *Hystrix africaeaustralis* | Cape porcupine | N | 1 |
|  | 4 | Rodentia | Sciuridae | *Paraxerus cepapi* | Tree squirrel | C | 2 |
|  | **5** | **Rodentia** | **Sciuridae** | ***Xerus inauris*** | **Southern African ground squirrel** | **D** | **4** |
|  | 6 | Rodentia | Muridae | *Acomys spinosissimus* | Spiny mouse | N | 1 |
|  | 7 | Rodentia | Muridae | *Mastomys natalensis sensu lato* | Natal multimammate mouse | N | 1 |
|  | **8** | **Rodentia** | **Muridae** | ***Aethomy chrysophilus*** | **Red veld rat** | **N** | **4** |
|  | 9 | Rodentia | Muridae | *Aethomys namaquensis* | Namaqua rock mouse | N | 2 |
|  | 10 | Rodentia | Muridae | *Otomys irroratus sensu lato* | Vlei rat | C | 1 |
|  | **11** | **Rodentia** | **Muridae** | ***Tatara leucogaster*** | **Bushveld gerbil** | **N** | **7** |
| **Class** | **No.** | **Order** | **Family** | **Scientific name** | **Common Name** | **Activity** | **# of roadkill detected over a 40-day period** |
| **Mammalia** | 12 | Rodentia | Muridae | *Rattus rattus* | Black rat | N | 1 |
|  | 13 | Primates | Galagidea | *Otolemur crassicaudatus* | Greater galago | N | 1 |
|  | 14 | Primates | Cercopithecidae | *Papio hamadryas* | Chacma baboon | D | 1 |
|  | 15 | Eulipotyphla | Soricidae | *Crocidura hirta* | Lesser red musk shrew | B | 1 |
|  | 16 | Chiroptera | Nycteridae | *Nycteris thebaica* | Egyptian slit-faced bat | N | 1 |
|  | **17** | **Chiroptera** | **Rhinolophidae** | ***Rhinolophus fumigates*** | **Ruppell’s horseshoe bat** | **N** | **4** |
|  | 18 | Carnivora | Hyaenidae | *Proteles cristatus* | Aardwolf | N | 1 |
|  | 19 | Carnivora | Viverridae | *Civettictis civetta* | African civet | N | 3 |
|  | 20 | Carnivora | Herpestidae | *Mungos mungo* | Banded mongoose | D | 1 |
|  | 21 | Carnivora | Canidae | *Octocyon megalotis* | Bat-eared fox | B | 2 |
|  | 22 | Carnivora | Canidae | *Canis mesomelas* | Black-backed jackal | B | 1 |
|  | 23 | Suiformes | Suidae | *Phacochoerus africanus* | Warthog | D | 1 |
|  | 24 | Ruminantia | Bovidae | *Aepyceros melampus* | Impala | D | 1 |
|  |  |  |  |  |  |  |  |
